# Supplementary material for: Thrombotic events and rebleeding after hemorrhage in patients taking direct oral anticoagulants for non-valvular atrial fibrillation
Source: PLoS One. 2021 Nov 29;16(11):e0260585. doi: 10.1371/journal.pone.0260585 (PMC8629319; doi:10.1371/journal.pone.0260585)
Supplement: Attachment — Submitted filename: Response To Reviewers.docx [file pone.0260585.s001.docx]

We greatly appreciate your careful reading of our manuscript and the helpful advice provided. We have revised our manuscript according to each of the comments we received.

We have responded to the issues raised in a point-by-point manner, as follows.

Editorial Board Member

Reviewer 1

1. This is a descriptive study or patients who have already bled. It cannot speak to incidence of first bleeding, risk factors for bleeding etc. The authors are generally cautious about this throughout the manuscript yet in the final conclusions the state that “the present study showed that GIB was common and serious among patients taking DOACs,” which would require knowing the incidence of bleeding among the 2005 patients mentioned in the methods section that were prescribed DOAC during this period. They then go on to state that “clinicians should endeavor to…” this study cannot speak to what should be done in practice. It is clear that patients who do not resume anticoagulation are generally of greater hemorrhagic and thrombotic risk than those who do not resume and that therefore, retrospective data cannot resolve the question over what clinicians should or should not do when facing a patient who had a major bleeding. Indeed, precisely because this is the case, a number of trials are ongoing randomizing resuming vs. not resuming anticoagulation in patients with intracranial hemorrhage (I am less sure about those with gastrointestinal bleed, but the question over confounders stands). While I am sympathetic to the idea that resuming should be the default position based on the best data we have, this study cannot be used to advise clinicians on what to do.

⇒We have deleted the following sentence from the original “Conclusions” section: “Clinicians should endeavor to resume anticoagulant therapy despite the high rebleeding rate when patients with major bleeding during DOAC therapy are encountered.”

2. A distinction between spontaneous and traumatic and surgical bleedings should be made throughout the manuscript. At least some are procedure-related based on the table (polypectomy). Nasal bleeding may be related to ENT examinations etc. I would judge these patients to have a lower re-bleeding risk (other than related to further procedures) and would rarely hesitate to recommend re-starting anticoagulation; at least it would give me less pause than a spontaneous major bleed would. This is important data.
⇒As mentioned, we should consider spontaneous bleeding and post-treatment complications separately. We have removed the data regarding hemorrhage due to medical procedures represented by “After polypectomy” from Table 2 and have performed the analyses again for a total of 96 patients.

3. I have some misgivings about splitting the cohort into GI bleeding and “other.” I worry that this was a post-hoc split the authors may have made after finding a statistical difference in severity or to have a “comparison” in what is really a descriptive study. The group of other is very heterogeneous to the extent that it becomes a meaningless category (what do nasal bleedings have to do with intracranial hemorrhages). I suggest focusing on the description of all hemorrhages, rather than on the comparison between these two groups, which should just be a side issue. In line with this, it is very hard to know what to do with figure 2, and I suggest removing it. Comparing these two groups, which are otherwise not comparable, is not useful. An informative comparison between these two very different groups would require taking into account many variables, both known and unknown, and this cannot be done in this small patient cohort. A crude comparison between patients who bleed from the GI tract and those who bleed from other sites can be deceiving.

⇒We agree that the comparison of these two groups was meaningless, so we have changed the structure of the text and Table 1, and have deleted the original Figure 2.

Reviewer 2

1. In the abstract, it would be very informative to actually give the numerical data for rebleeding and thrombosis speparately for those that had anticoagulation resumed and those that did not. As currently writen it is not clear what these figures for complications after the initial bleeding episode represent.

⇒We have added the number of rebleeding and thrombosis events for those patients who resumed anticoagulants and for those from whom anticoagulants were withheld in the abstract.

2. The flow chart explaining subject flow through the study needs more clarity. The reasons for exclusion need to be given and the disposition of subjects made clearer. Simialrly from over 2000 patients with atrial fibrillation there appear to be no deaths during the follow-up period. Is this really the case?

⇒We have added exclusion criteria in the “Study subjects” section. Details of each exclusion criterion and the corresponding numbers of patients are clarified in the revised Figure 1. While we obtained 2005 patients from the initial prescription list of DOAC, we actually followed-up 172 patients who were prescribed DOAC for NVAF and experienced clinically relevant bleeding. Ten patients were unable to be followed-up during the follow-up period (Fig. 1), six of whom died.

3. Although the authors have used the data they have available. The paper should contain a formal statement about samoke size and power calculation related to the planned primary outcome, this will enable the reader to determine if the study is really able to answer the question that the authors have raised. The methods section needs to be clearer about what the primary hypothesis being examined in this study is.

⇒Although it may differ from hypothesis, the purpose of this study was stated at the end of the “Introduction” section, as follows: “The present study aimed to clarify the occurrence of rebleeding and thrombotic events in post-bleeding patients and the relationships with clinical factors such as resumption or discontinuation of anticoagulation therapy.”

4. The discussion about subjects excluded from the study, currently placed in data collection, more appropriately belongs in the study design and subjects section.

⇒We have moved the description of exclusion criteria to the “Study subjects” section. Details for each exclusion criterion and corresponding numbers of patients have been clarified in Figure 1.

5. Although the focus of this study is on outcomes after the 1st bleeding episode, it is unfortunate given the comprehensive follow up that Table 1 does not include the data for the group that had no bleeding, to allow clearer comparsion.

⇒We respect your opinion, but the main focus of this study was **the course of** **bleeding patients** taking DOAC with resumption or discontinuation. This is why we did not describe data for non-bleeding subjects.

6. The data of most interest to clinicians will be the outcomes after the 1st bleed, and the differentiation between those restarted and those without anticoagulation. Hence burying the combined data at the bottom of a summary table 3 is rather unhelpful. These data should be represented separately, although the numbers are small.

⇒Tables 5 and 6 provide data regarding rebleeding and cardiovascular events, in total and divided into subgroups with resumption or withholding of anticoagulants.

7. The authors have provided a lot of data on the co-morbidities of the group post-index bleeding. I am not sure such granular detail helps at all, especially as the authors have not clearly defined what they consider the inclusions for each of their criteria. Including the data on HAS-BLED scores for the rebleeding and non-rebleeding groups is essntial. This would allow greater generarisation.

⇒We have added median HAS-BLED scores for each of the rebleeding and non-rebleeding groups to Table 5.

8. The overall rebleeding rate seems comparable to other studues, again the authors should be able to estimate from HAS-BLED scores whether the rebleeding rate in their cohort is higer or lower than anticipiated.

⇒The correlation between HAS-BLED scores and rebleeding has been analyzed statistically. HAS-BLED scores tended to be higher in rebleeding patients, but remained non-significant. We have included this information in the revised text (page 16).

9. The authors seem rather over reliant in "p" values to assess differences between groups. It would be more appropriate to repesent these differences a odds ratio/hazaed ratio with 95% confidence intervals. Hence tables 5 and 6 should have these data included For clarity, it would be reasonable to leave out the OR/HR where there is clearly no difference but where the paper cites a "p" value the OR/HR with confidence interval is the correct way to represent this.

⇒According to this comment, we have added adjusted odds ratios with 95% confidence intervals to Tables 5 and 6.

Reviewer 3

1. It would be very useful to present more details as to how the anticoagulant drug was restarted. In Table 3 we are informed that 45 pts restarted same drug and 45 changed medication. I believe that this is very vague. We should know if the dose was reduced, if the NOAC was changed (and also from which agent to which agent the change was made) or if warfarin was recommended. A table with the number of pts who remained on same NOAC dose, who restarted lower dose of NOAC and who switched NOAC (or went back to warfarin) would do.

⇒We have provided details of the administration of anticoagulants after initial bleeding on Table 4.

2. A large number of pts was also receiving concomitantly antiplatelet drugs. And it appears that AFTER the bleeding episode many (or all?) continue on them. For example 28 pts were on aspirin (and 17 on P2Y12) before bleeding (table 1) and, surprisingly, 28 remain on aspirin (and 17 on P2Y12) after bleeding (table 5 and 6). Similarly the same number of pts is on PPI before and after bleeding (55 pts) which is odd. The common practice in AF pts who bleed is to withdraw the antiplatelet therapy first rather than stop the NOAC.

⇒Since most subjects in this study had a history of atherosclerotic diseases such as myocardial infarction and cerebral infarction, almost all patients had to continue antiplatelet therapy. Incidentally, we received advice from other reviewers to remove the comorbidities from Tables 5 and 6, and we have followed that advice.

3. The rate of MACCE in this group of 102 pts is 2.5%/year. It would be interesting to have the rate for the 2005 pts overall (to confirm that pts who bleed are a high risk group)

⇒We respect your opinion, but we considered that the main focus of this study was **the course of** **bleeding patients** according to the resumption or discontinuation of DOACs. That is why we have not provided data for non-bleeding subjects.

4. Accordingly the above issues should be commented in the discussion.

⇒We have added discussion of these issues, as requested.

Reviewer 4

Introduction
1. DOACs have been approved for clinical use for over 10 years (i.e. no longer "recent"). In light of this, what additional information does this study provide beyond existing literature?
⇒As mentioned in the last part of the Introduction section, knowledge of the clinical course after bleeding during DOAC therapy remains limited. Hence the incidences of rebleeding and thrombosis, along with factors associated with the development of such adverse events, were considered to represent important and essential information.

2. Anticoagulant discontinuation after bleeding is associated with a higher risk of thrombosis and death, but studies were limited by baseline confounding and likely differences in prognosis among patients who resumed versus those who did not resume anticoagulation after bleeding

⇒To minimize the effects of confounders, differences in ratios were evaluated by linear correlation model using inverse probability weighting methods. Since the method of statistical analysis has been changed, the text has been amended accordingly in the Statistics section.

3. it would be helpful to provide specific objectives for readers. As written, "clarify the course after bleeding" is vague.
⇒We have modified the last paragraph of the Introduction section, as follows: “The present study aimed to clarify the occurrence of rebleeding and thrombotic events among post-bleeding patients and the relationships with clinical factors such as resumption or discontinuation of anticoagulation therapy”. We have also revised the Abstract to provide a detailed description regarding this content.

Methods
4. In general, additional details regarding the methods are required.

⇒We have added exclusion criteria for subjects of this study in the “Study subjects” section. As mentioned in the response to Comment 2, the method of statistical analysis has been changed and described in detail. HAS-BLED and CHADS2 scores were newly evaluated as factors for rebleeding and thrombosis, respectively, and this content has been added to the “Data collection” section.

5. Further, some results are included in the methods section (i.e. 2005 patients)

⇒The numbers of initially extracted patients and finally selected subjects have been moved from the “Methods” to the “Results”.

6. Please list specific inclusion/exclusion criteria at the beginning of the Study Population section. Patients with AFib? Any dose of DOAC? Why were patients receiving DOACs for less than one month excluded?

⇒We have described the exclusion criteria in the “Study subjects”. The reason for excluding subjects less than a month after starting DOACs was that such cases may have included patients who had not made another outpatient visit after starting DOAC and medication status thus could not be confirmed.

7. Were these patients identified by outpatient prescriptions?

⇒Yes, all subjects were outpatients. Inpatient status was one of the exclusion criteria.

8. Did patients have both reliable inpatient and outpatient follow-up?

⇒Yes, we definitely followed-up all subjects. Patients who were unable to complete follow-up after bleeding were excluded.

9. What does "able to be followed up" mean? This indicates that they may not have captured all potential patients. What about patients who weren't able to be followed? It would be important to document loss to follow-up.

⇒We have deleted the sentence including “able to be followed up”. We have clarified that ten patients could not be followed and were thus excluded from the analysis (Fig 1).

10. Please clarify the reason BARC was selected as opposed to ISTH definitions which are commonly used in this population

⇒The reason we selected BARC was that BARC is better than the ISTH definition for identifying mild but clinically meaningful bleeding.

11. What does "clinical course was examined every month" mean?

⇒This sentence means that we checked on the clinical progress monthly based on the medical records. As we considered this comment unnecessary comment, we have deleted it.

12. Did you collect information about DOAC dosing?

⇒We have shown details for the administration of anticoagulants after initial bleeding in Table 4.

13. No adjustment for baseline confounding was done (e.g. by regression). Some justification should be provided about why associations were not explored using regression analysis.

⇒To minimize the effects of confounders, differences in ratios were evaluated by linear correlation model using inverse probability weighting methods and have added adjusted odds ratios with 95% confidence intervals to Tables 5 and 6.

14. What was the duration of follow-up?
⇒Follow-up was continued for a total of 242.6 patient-years, as shown in Table 3.

Results
15. the rate of clinically relevant bleeding in this cohort seems low (102 out of 2005 patients) over 6 years

⇒This may be because we only followed-up patients with non-valvular atrial fibrillation, not all patients prescribed DOACs.

16. Table 1 should indicate the type of bleeding at cohort entry. Not sure why GI bleeding and "Other bleeding" were separated out into columns. Please include individual DOACs on separate lines (as opposed to D/R/A/E)

⇒We have completely modified Table 1. Gastrointestinal bleeding and other bleeding were not separated and each DOAC was listed on a separate line.

17. What is meant by "upper GIB tended to be more serious"? Hospitalization? ICU admission? Hemodynamic instability?

⇒We have added more detailed explanations about “more serious” and have corrected the sentence on page 8 as follows: “Upper GIB was associated with a higher rate of BARC type 3 or more, meaning that upper GIB tended to be more serious”.

18. "Tendency to become serious" is vague

⇒We have deleted the sentence regarding the “tendency to become serious”. Other reviewers pointed out that the distinction between GIB and other bleeding seems arbitrary to create a statistical differences, and “other bleeding” was a meaningless category, because the different types of other bleeding were not related to each other. We have therefore also deleted all descriptions about comparisons between GIB and other bleeding.

19. "Little difference was seen between patients with GIB and those with other bleeding" with respect to comorbidities is also vague

⇒For the reasons described in Comment 18, the description regarding the comparison of GIB and other bleeding has been deleted.

20. Any differences in dose for restarting anticoagulation?

⇒We have shown details for the administration of anticoagulants after initial bleeding in the revised Table 4.

21. Did severity of bleeding or site of bleeding influence therapy?

⇒This study did not examine the relationship between severity of bleeding or site of bleeding and therapeutic strategy, but only described how many patients resumed or had DOAC anticoagulants withheld after bleeding on Table 4.

22. Did you capture all-cause death within your cohort? How was cardiac death adjudicated?

⇒We captured all causes of death, and defined cardiac death as death due to exacerbation of heart failure and sudden cardiac death.

23. When did re-bleeding occur?

⇒Median period from initial bleeding to rebleeding was 18 months (range, 1–60 months). The result has been described in Table 5.

24. Remove comorbidites from Tables 5 and 6

⇒We have removed the comorbidities from Tables 5 and 6.

25. What about loss to follow-up?

⇒We used the term “dropout cases” in the “Data collection” section. The definition of dropout was described as the patient stopping visits to our institution for >6 consecutive months without a documented reason.

Discussion
26. the conclusions are overstated. It is not clear how the authors showed conclusively that resumption of anticoagulation was not associated with rebleeding as it is stated and based on the analyses conducted

⇒We have deleted the following sentence of “Conclusions” section: “Clinicians should endeavor to resume anticoagulant therapy despite the high rebleeding rate when patients with major bleeding during DOAC therapy are encountered.”

27. what does this study add to existing literature?

⇒⇒As mentioned in the last part of the Introduction section, the clinical course after bleeding during DOAC therapy remains limited. The incidences of rebleeding and thrombosis, as factors associated with the development of adverse events, were thus considered to represent important and essential information.

28. The COMPASS trial showed that prophylactic PPI did not influence bleeding (factorial randomization)

⇒In the COMPASS trial, subjects allocated to the pantoprazole group originally did not require PPI administration, and a lot of mild cases might have been registered.

29. Anticoagulant related GI bleeding is more than "troublesome" with mortality rates of ~10% at 30 days in some cohorts

⇒We have changed “troublesome” to “life-threatening”.

30. Clinical recommendations about the timing of treatment re-initiation is beyond the scope of this paper and remains highly uncertain
⇒We have deleted the following sentence (page 14, line 15), “Resuming anticoagulant administration within 1 month appears desirable as a response after hemorrhagic events, particularly GIB”.
