# Supplementary material for: Thrombotic events and rebleeding after hemorrhage in patients taking direct oral anticoagulants for non-valvular atrial fibrillation
Source: PLoS One. 2021 Nov 29;16(11):e0260585. doi: 10.1371/journal.pone.0260585 (PMC8629319; doi:10.1371/journal.pone.0260585)
Supplement: Attachment — Submitted filename: Response to Reviewers.docx [file pone.0260585.s002.docx]

We greatly appreciate your careful reading and meaningful advice. We have revised our manuscript according to the comments provided.

We have answered the issues raised point-by-point, as follows.

Editorial Board Member

Reviewer 1

1. The authors have removed a comment that spoke to the clinical decisions that physicians should make based on this retrospective study, given the potential biases. However, another such comment is present in the current version. Page 14 line 246 “clinicians should resume anticoagulants after bleeding events….” I still think this study (or any of the other studies published, which are all retrospective and/or observational) cannot be used to reach this conclusion. I suggest removing this statement.

⇒We have deleted the sentence on page 14 line 246 that you pointed out.

2. Most importantly for clarity I think the authors should use consistently bleeding for the first bleeding (the one leading to study entry) and rebleeding for subsequent hemorrhages. While they occasionally do, this is not consistent (e.g., section title “Bleeding and thrombosis after initial bleeding” would be “Rebleeding and thrombosis…”), page 9 line 163 “…11 developed rebleeding”, table 3 (title and table itself) etc . I think this would help follow the text more easily.
⇒We have corrected all points which were appropriate to use “rebleeding”.

3. Methods. Page 4 lines 76-78 and page 5 lines 92 -93 are redundant (same exclusion criteria are mentioned).

⇒We have removed the sentence on page 5 lines 92-93 that described regarding the exclusion criteria.

4. Statistics section fail to mention most of the analyses conducted.

⇒As you mentioned, it was not fully explained regarding statistics. We have added a description in “statistics” section.

5. Table 1 title is confusing as “baseline” and “at index bleeding” seem contradictory (baseline would generally mean at the time of starting anticoagulation).

⇒We have changed Table 1 title as “Characteristics of study subjects at index bleeding” and also subheading on page 6 lines 124 as follows, “Characteristics of subjects at index bleeding”.

6. Tables 5 and 6 include “E8” on several occasions in the aOR column. Meaning is unclear. Does that mean 10 raised to the power of 8? This seems unplausible but in any event meaning should be cleared up.

⇒We have corrected E7, E8 and E10 to 10^7^, 10^8^ and 10^10^.

Reviewer 2

1. Amend the title to include......for non-valvular atrial fibrillation. The study specifically focuses on this group of patients taking DOACs, and although the results are likely to have some generalisability, the risk stratification for other indications for DOACs may be different.

⇒We have changed the title to “Thrombotic events and rebleeding after hemorrhage in patients taking direct oral anticoagulants for non-valvular atrial fibrillation”.

2. The authors give rates of bleeding events and thrombotic events per year, in the Results section. Given the small cohort and limited number of events (especially thrombotic) the confidence intervals around these rates are likely to be high. By just giving a single figure, this suggests that rates of bleeding a nearly three times higher than thromboembolism. I doubt that the data are rich enough to say that for certain. Please include some marker of the range or confidence intervals around these calculations of event rates.

⇒Did you pointed out the matter about Table 3? Indeed, we gave rates of bleeding in 4.5%/year, cardiac and cerebrovascular event in 2.5%/year, and systemic thrombosis in 1.6%/year. These rates were simply calculated by dividing the number of events by the observation period. It may be a lack of my understanding, but we wonder if we can calculate the 95% confidence interval of event rates.

Reviewer 3

1. The paper is now more informative. My only concern is again related to the use of antiplatelet drugs. It is not clear if rebleeding was mainly recorded in pts who remained on combination therapy. It is unfair to attribute a bleeding (or re-bleeding) on DOAC rather than on antiplatelet if this was also given. At least a comment in the discussion is needed.

⇒We have evaluated the influence of concomitant antiplatelet on the risk of rebleeding by generalized linear model using propensity score weighting by inverse probability weighting method, and the results were shown in Table 5. There was no significant difference statistically, which described on page 11 lines 182-183.
