# Supplementary material for: Thrombotic events and rebleeding after hemorrhage in patients taking direct oral anticoagulants for non-valvular atrial fibrillation
Source: PLoS One. 2021 Nov 29;16(11):e0260585. doi: 10.1371/journal.pone.0260585 (PMC8629319; doi:10.1371/journal.pone.0260585)
Supplement: Attachment — Submitted filename: Response to Reviewers.docx [file pone.0260585.s003.docx]

We greatly appreciate your careful reading and meaningful advice. We have revised our manuscript according to the comments provided.

We have answered the issues raised point-by-point, as follows.

Reviewer 3

1. I think that a brief comment in the discussion should adress the fact that some pts restarted not only the NOAC but the antiplatelet as well.

⇒We have added a comment regarding the fact that the majority of rebleeding patients resumed combined use of antithrombotic agents. We have described on page 14 lines 240-243 as follows; it is to be noted that all cases of rebleeding resumed anticoagulation therapy, and more than half of them were also on antiplatelet drugs concurrently. The necessity for combined use of antithrombotic drugs may be considered carefully in patients with a history of bleeding.
